# Frequency, underdiagnosis, and heterogeneity of epidermal growth factor receptor exon 20 insertion mutations using real‐world genomic datasets

**DOI:** 10.1002/1878-0261.13327

**Published:** 2022-11-28

**Authors:** Santiago Viteri, Anna Minchom, Lyudmila Bazhenova, Sai‐Hong Ignatius Ou, Joshua M. Bauml, Scott A. Shell, Michael Schaffer, Junchen Gu, Jennifer B. Rose, Joshua C. Curtin, Parthiv Mahadevia, Nicolas Girard

**Affiliations:** ^1^ UOMI Cancer Center Clínica Mi Tres Torres Barcelona Spain; ^2^ Drug Development Unit Royal Marsden/Institute of Cancer Research Sutton UK; ^3^ University of California San Diego CA USA; ^4^ Chao Family Comprehensive Cancer Center University of California Irvine School of Medicine Orange CA USA; ^5^ Perelman School of Medicine at the University of Pennsylvania Philadelphia PA USA; ^6^ Guardant Health Redwood City CA USA; ^7^ Janssen R&D Spring House PA USA; ^8^ Institut Curie Institut du Thorax Curie‐Montsouris Paris France; ^9^ Present address: Janssen R&D Spring House PA USA

**Keywords:** EGFR, Exon 20 insertion mutations, NGS, NSCLC, PCR

## Abstract

Epidermal growth factor receptor (EGFR) exon 20 insertion mutations (ex20ins) account for ≤ 12% of all EGFR‐mutant nonsmall cell lung cancers. We analysed real‐world datasets to determine the frequency of ex20ins variants, and the ability of polymerase chain reaction (PCR) and next‐generation sequencing (NGS) to identify them. Three real‐world United States NGS databases were used: GENIE, FoundationInsights, and GuardantINFORM. Mutation profiles consistent with in‐frame EGFR ex20ins were summarized. GENIE, FoundationInsights, and GuardantINFORM datasets identified 180, 627, and 627 patients with EGFR ex20ins respectively. The most frequent insertion region of exon 20 was the near loop (~ 70%), followed by the far loop (~ 30%) and the helical (~ 3–6%) regions. GENIE, FoundationInsights, and GuardantINFORM datasets identified 41, 102, and 96 unique variants respectively. An analysis of variants projected that ~ 50% of EGFR ex20ins identified by NGS would have been missed by PCR‐based assays. Given the breadth of EGFR ex20ins identified in the real‐world US datasets, the ability of PCR to identify these mutations is limited. NGS platforms are more appropriate to identify patients likely to benefit from EGFR ex20ins‐targeted therapies.

## Graphical abstract

Three next‐generation sequencing (NGS) databases (GENIE, FoundationInsights and GuardantINFORM) were analyzed to estimate the frequency of exon 20 insertion (ex20ins) variants in epidermal growth factor receptor (EGFR)–mutant non‐small cell lung cancer (NSCLC). Results indicate ~ 50% of ex20ins identified by NGS would have been missed by PCR and the near loop is the most frequent insertion site.

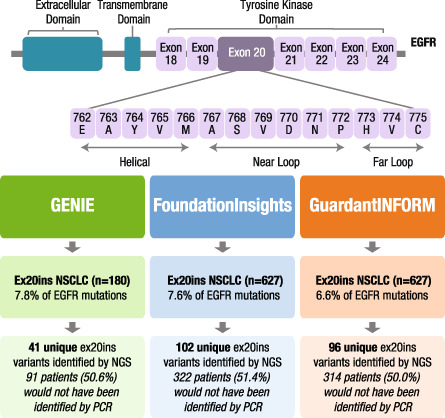

**Abbreviations**
AA: amino acid
EGFR: epidermal growth factor receptor
EGFRm: epidermal growth factor receptor‐mutant
ex20ins: exon 20 insertion mutation
GENIE: Genomics Evidence Neoplasia Information Exchange
NGS: next‐generation sequencing
NSCLC: nonsmall cell lung cancer
ORR: overall response rate
PCR: polymerase chain reaction
TKI: tyrosine kinase inhibitor

## 1 Introduction

Mutations in the epidermal growth factor receptor (EGFR) are a key oncogenic driver in nonsmall cell lung cancer (NSCLC). The common mutations, exon 19 deletions and L858R substitution in exon 21, are detected in 85–90% of all EGFR‐mutant (EGFRm) NSCLCs [1]. The third most common type of EGFRm NSCLC is comprised of exon 20 insertion mutations (ex20ins), which are characterized by in‐frame insertions or duplications typically clustered within or after the C‐helix in the EGFR kinase domain [1]. The reported frequency of EGFRm NSCLCs harbouring ex20ins is between 1% and 12% [2]. This broad range likely reflects the molecular heterogeneity of EGFR ex20ins and the limitations of available molecular assays to identify them [3].

Unlike common mutations, EGFR ex20ins are less sensitive to EGFR tyrosine kinase inhibitors (TKI) because the insertions create steric hindrance at the TKI‐binding site [4]. The reported overall response rate (ORR) to approved EGFR TKIs is between 0% and 28% compared with response rates of 60–80% in patients with the common mutations [1, 5]. Further, two real‐world studies found overall survival was significantly lower (by ~ 10 months) for patients with ex20ins compared with those who have common EGFR mutations (16.2 versus 25.5 months [6] and 24.3 versus 35.4 months [7]). However, the Y764insFQEA variant has shown sensitivity to EGFR TKIs, suggesting that differential response to TKI treatment may be associated with the type and site of ex20ins [3, 5]. Additionally, rates of sensitizing EGFR ex20ins vary geographically [8]; however, studies have found antitumour activity across ex20ins sites with amivantamab [9] and similar ORRs to mobocertinib in the near and far loops of exon 20 [10].

Investigational therapies for the treatment of EGFR ex20ins NSCLC are in development [5]. Amivantamab, an EGFR‐MET bispecific antibody, and mobocertinib, an ex20ins‐specific TKI, were recently approved by the FDA (and also the EMA for amivantamab) for the treatment of patients with EGFR ex20ins NSCLC, as detected by an FDA‐approved test, whose disease progressed on or following platinum‐based chemotherapy [9, 10, 11, 12]. Therefore, the identification of tumours bearing EGFR ex20ins is important in guiding treatment decisions, and universal molecular testing should be able to accurately and comprehensively detect the wide range of variants that have been identified thus far. Real‐time polymerase chain reaction (PCR) and next‐generation sequencing (NGS) are two molecular methods commonly used to identify mutations in the EGFR gene [13, 14]. The commercially available PCR‐based assays detect ‘hot spot’ mutations and are designed to identify only a subset of known ex20ins. In contrast, NGS has the ability to detect multiple types of genetic mutations over a specified region, including any new mutations not identified previously [13, 14], and is therefore expected to provide more complete coverage of EGFR ex20ins than PCR‐based assays. The use of NGS has also steadily increased over the past decade [13], and the updated guidelines from the IASLC support the use of sequential or concurrent NGS tissue and liquid biopsy analyses [15]. In this report, three real‐world US NGS genomic databases were analysed to determine the frequency of EGFR ex20ins and to compare the ability of PCR versus NGS‐based testing methods to identify these mutations.

## 2 Materials and methods

### 2.1 US genomic NGS databases

The American Association for Cancer Research Project Genomics Evidence Neoplasia Information Exchange (GENIE) database is a public registry of real‐world NGS genomic data collected from participating cancer centres [16]. In this analysis, NGS data extracted from the GENIE database, version 8.5, were limited to the 13 participating US institutions covering a total of 80 015 patients. DNA sequencing of tumour biopsy samples occurred between 2012 and 2019.

The FoundationInsights (Foundation Medicine, Cambridge, MA, USA) real‐world US NGS database, version MI20200428, based on the FoundationCore knowledgebase of patient genomic profiles, spans over 150 cancer types. At the time of analysis (data cut‐off of 28 April 2020), the database contained information for 315 688 samples from tumour biopsies.

The GuardantINFORM database contains real‐world US NGS data collected from Guardant360 CDx liquid biopsy tests from over 174 087 patients (Guardant Health, Inc., Redwood City, CA, USA). The database query covered 1 February 2015 through 30 June 2021.

Given the limited scope of this study, information, such as demographics, clinical staging, treatments, and outcome variables, was not available. Only certain demographics (e.g. age, race, and gender) could be obtained from the GENIE database.

### 2.2 Data analysis

Exon 20 of the EGFR gene encompasses nucleotides that translate to amino acid (AA) positions 762–823. Mutation profiles from all three real‐world datasets were evaluated for alterations (duplications and deletion/insertion) consistent with in‐frame ex20ins of the EGFR gene. Ex20ins were summarized based on the resulting AA changes and were aggregated into frequencies per alteration. Coverage of ex20ins from commercially available PCR‐based assays, therascreen EGFR RGQ PCR Kit version 2 (Qiagen, Hilden, Germany) and cobas EGFR v.2 (Roche, Basel, Switzerland) were obtained from their instructions for use (Table S1).

## 3 Results

**Fig. 1:**
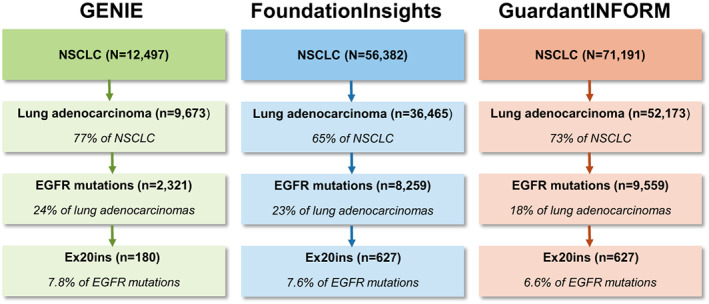
Patient selection across three real‐world US databases. NGS data were extracted from the GENIE, FoundationInsights, and GuardantINFORM databases to identify patients with lung adenocarcinoma who harboured ex20ins. Data from the GENIE database were limited to those from participating US institutions. EGFR, epidermal growth factor receptor; ex20ins, exon 20 insertion mutation; NSCLC, nonsmall cell lung cancer.

To determine the real‐world frequency of EGFR ex20ins, we analysed data from three NGS genomic datasets that included patients with NSCLC: GENIE (*N* = 12 497), FoundationInsights (*N* = 56 382), and GuardantINFORM (*N* = 71 191). Among patients with NSCLC, EGFRm lung adenocarcinoma was diagnosed in 24% (GENIE), 23% (FoundationInsights), and 18% (GuardantINFORM) of patients (Fig. 1). Of note, the percentage of patients with EGFRm NSCLC from the FoundationInsights and GuardantINFORM databases reflect the ordering patterns of physicians and are not intended to represent the expected frequency of these patients in an unbiased US‐based patient population. Among the 9673 patients with NSCLC adenocarcinomas in the GENIE database, the median age was 67 years old, most patients were female (60%) and the majority were White (77%), followed by African American/Black (7.6%) and Asian (6.9%). Demographics were not included from FoundationInsights and GuardantINFORM for this study.

**Fig. 2:**
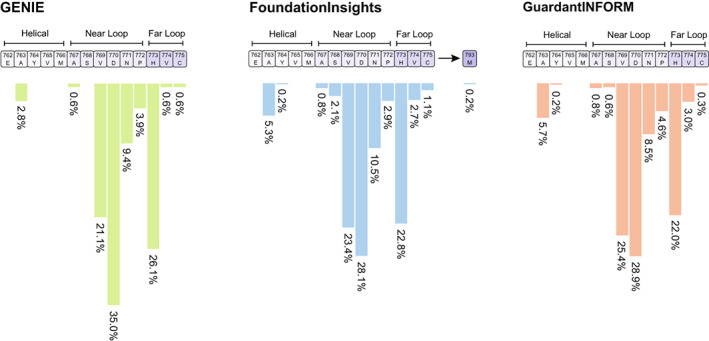
Frequency by site of insertion in exon 20 of the EGFR. The distribution of ex20ins by site of AA insertion from the GENIE (green; *N* = 180), FoundationInsights (blue; *N* = 627) and GuardantINFORM (orange; *N* = 627) datasets. Percentages are calculated using number of patients with EGFR ex20ins lung adenocarcinoma as the denominator. EGFR, epidermal growth factor receptor; ex20ins, exon 20 insertion mutation.

The GENIE, FoundationInsights, and GuardantINFORM datasets identified 180, 627, and 627 patients with EGFR ex20ins, respectively, which accounted for ~ 7–8% of all EGFRm lung adenocarcinomas (Fig. 1). Across all three datasets, ~ 70% of EGFR ex20ins were found in the near loop region (AA 767–772), ~ 30% in the far loop region (AA 773–775), and ~ 3–6% in the helical region (AA 762–766). The most frequent sites of insertion identified in all three datasets were located at AA 769 (21.1–25.4%), AA 770 (28.9–35.0%), and AA 773 (22.0–26.1%; Fig. 2).

**Fig. 3:**
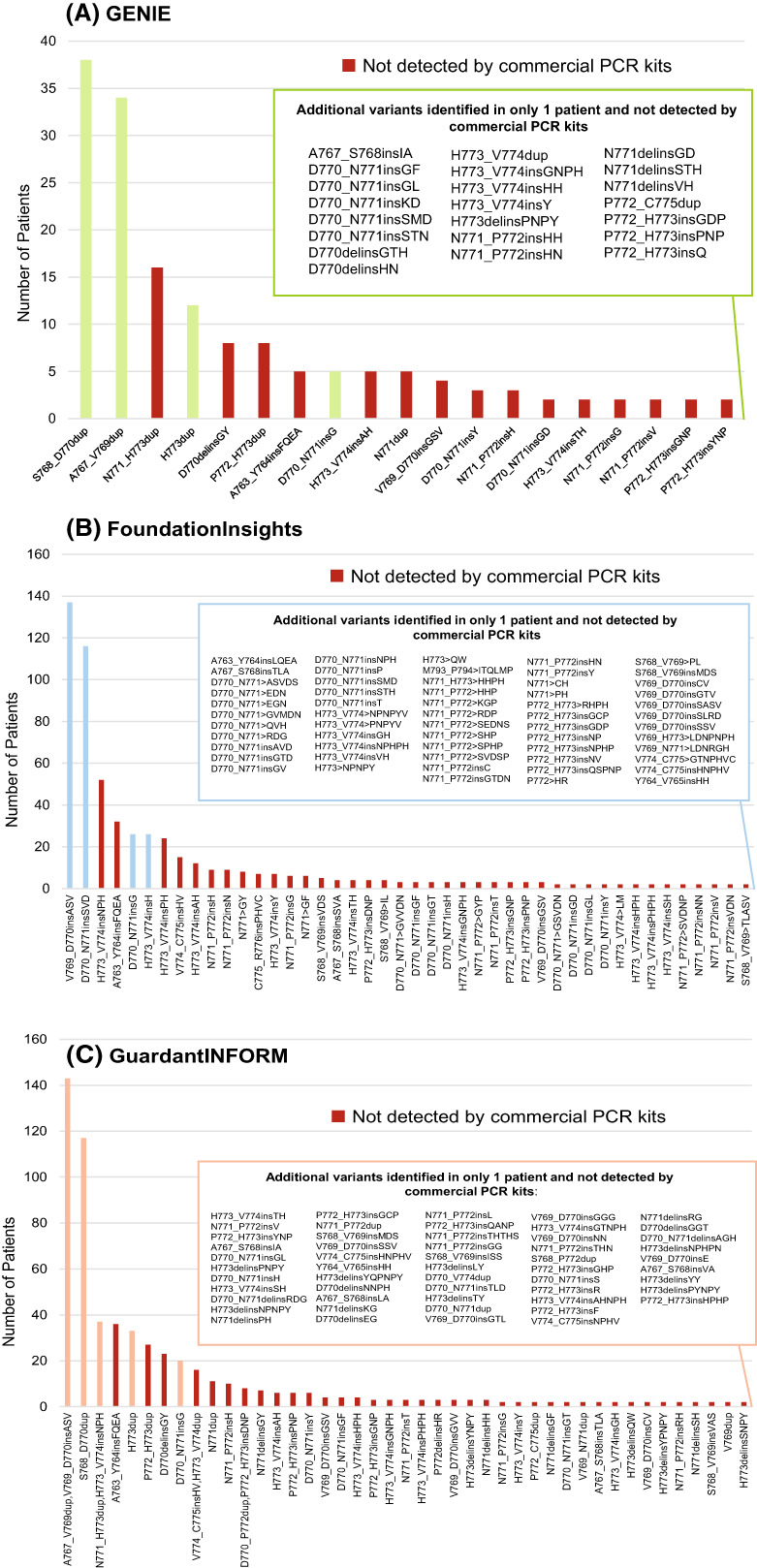
EGFR ex20ins variants identified from the (A) GENIE (*N* = 180), (B) FoundationInsights (*N* = 627), and (C) GuardantINFORM (*N* = 627) datasets. Ex20ins variants identified in two patients or more are included in the bar chart. Ex20ins variants identified in only one patient and not detected by commercial PCR‐based assays are listed in the box (inset). Red bars denote ex20ins variants that would not have been detected using commercial PCR‐based assays. EGFR, epidermal growth factor receptor; ex20ins, exon 20 insertion mutation; PCR, polymerase chain reaction.

The molecular heterogeneity of EGFR ex20ins was evident, with 41, 102, and 96 unique variants identified from the GENIE, FoundationInsights, and GuardantINFORM datasets respectively (Tables S1–S3). If the coverage for EGFR ex20ins from commercial PCR‐based assays is applied to the GENIE dataset, only 89 of the 180 patients identified by NGS would have been identified, thereby missing 50.6% of all cases (Fig. 3A). In the FoundationInsights dataset, commercial PCR‐based assays would have identified only 305 of the 627 patients identified by NGS, missing 51.4% of all EGFR ex20ins cases (Fig. 3B). Similar findings were observed with the GuardantINFORM dataset, where PCR‐based assays would only have identified 313 of the 627 patients with EGFR ex20ins, missing 50.0% of patients (Fig. 3C).

Across all three datasets, only 23 EGFR ex20ins variants were found, underscoring the diversity of this class of mutations. Variants that were found exclusively in each dataset accounted for 24% (10/41) of the GENIE dataset, 50% (51/102) of the FoundationInsights dataset, and 47% (45/96) of the GuardantINFORM dataset (Tables S2–S4), suggesting a considerable degree of genomic heterogeneity among real‐world US NGS databases.

## 4 Discussion

In this real‐world analysis using three US NGS genomic datasets, EGFR ex20ins variants account for ~ 1% of all NSCLC cases in each of the three databases. The estimated frequency of ex20ins was ~ 7–8% of all EGFRm lung adenocarcinomas, which is consistent with the range (up to 12%) reported in the literature from other US‐based databases [13] and globally [2, 17, 18, 19, 20]. The near loop was the most frequent insertion region of EGFR ex20ins, where ~ 70% of all insertions were found. Insertions in the helical region, where sensitivity to EGFR TKIs has been reported [3, 5], were uncommon. Importantly, the frequency of EGFR ex20ins observed in datasets that utilize NGS on tumour biopsies (GENIE and FoundationInsights) was generally consistent with that observed using liquid biopsies (GuardantINFORM), suggesting that both testing methods could accurately identify the diverse range of these mutations.

Over 80 distinct EGFR ex20ins variants have been identified [2]. This result is consistent with our study, where a large number of unique EGFR ex20ins variants were identified from the real‐world NGS datasets, ranging from 41 variants in the GENIE dataset to 102 in the FoundationInsights dataset. Twenty‐three EGFR ex20ins variants, which included those most frequently observed, were common to all three datasets. The genomic heterogeneity and breadth of EGFR ex20ins identified in these real‐world datasets emphasize the need for an accurate and comprehensive method to detect these mutations.

Although PCR‐based assays have a quicker turn‐around time, they have limited capacity for assessing multiple genes and variant types [14]. Real‐world, retrospective studies have shown higher rates of detection of EGFR ex20ins with NGS compared with PCR [21] and similarly have suggested a high rate of missed EGFR ex20ins mutations with PCR testing versus NGS [22]. In this analysis, of the EGFR ex20ins variants identified in real‐world NGS databases, commercial PCR‐based assays would have detected only four of the variants, missing ~ 50% of all cases; however, in this population of patients, there are now two commercially available NGS tests (Oncomine Dx Target Test and Guardant360 CDx) approved as companion diagnostics for amivantamab and mobocertinib. Given the limited capacity of PCR‐based assays to detect multiple genes and variants, sequential testing may be needed to cover the full range of potential biomarkers. This additional workflow would lead to increased costs, require larger tumour samples, and delay time to treatment [14, 23]. In contrast, NGS offers comprehensive testing, with the ability to detect all relevant biomarkers (and yet‐to‐be‐identified mutations) in a single test, either in tissue or liquid biopsies. This can reduce the need for re‐biopsy, decrease the time to obtain test results, and potentially provide cost savings by negating the need for sequential single‐gene tests [23]. It should be noted that there are also limitations to NGS, such as difficulty interpreting novel variants, which may require the implementation of molecular tumour boards, lack of knowledge of the appropriate number of genes to be screened, and how to handle discordancy between commercially available tests run in parallel [24], the latter of which is not an unexpected entity as a recent review on liquid biopsies had also found poor concordance in ctDNA of prostate cancer samples between multiple assays [25].

The results of our study should be interpreted within its limitations. Potential confounders were not assessed due to the lack of demographic information excluded from all three databases.

## 5 Conclusions

In this analysis, the real‐world US frequency of ex20ins was ~ 7–8% of all EGFRm lung adenocarcinomas. With up to 102 unique variants identified, the ability of PCR‐based assays to comprehensively identify patients with these mutations is limited. The identification of all EGFR ex20ins variants will be important when implementing dedicated targeted therapies in the first‐line setting. With several investigational therapies in development and the recent approval of amivantamab and mobocertinib, identifying patients who harbour these mutations with NGS‐based testing methods will be important to ensure appropriate patient access to new targeted therapies.

## Conflict of interest

**SV** reports consulting and/or advising for AbbVie, Bristol Myers Squibb, Roche, Takeda, AstraZeneca, and MSD; speakers bureau fees from Bristol Myers Squibb, MSD, Roche, and AstraZeneca; and travel expenses from Roche, OSE Pharma, Bristol Myers Squibb, Merck, Merck Serono, Puma Biotechnology, and Janssen Cilag. **AM** reports teaching and/or serving on an advisory board for Chugai, Janssen, Merck, Novartis, Faron, and Bayer; and expenses from LOXO and Amgen. **LB** reports consultative services for Daiichi Sankyo, Boehringer Ingelheim, Janssen, Bristol Myers Squibb, Merck, Novartis, and Regeneron; and research funding from BeyondSpring. **S‐HIO** reports stock ownership of Turning Point Therapeutics, and Elevation; speaker honoraria from Merck, Roche/Genentech, AstraZeneca, Takeda/ARIAD, and Pfizer; and advisory fees from Roche/Genentech, AstraZeneca, Takeda/ARIAD, Pfizer, Foundation Medicine, Spectrum, X‐Covery, Janssen/Johnson & Johnson, and Daiichi Sankyo. **JMB** is a current employee of Janssen R&D and reports previous research/grant support from Merck, Clovis, Carevive Systems, Novartis, Bayer, Janssen, AstraZeneca, Takeda, and Carisma Therapeutics, and consultative services for Clovis, Bristol Myers Squibb, AstraZeneca, Celgene, Boehringer Ingelheim, Janssen, Merck, Guardant Health, Genentech, Takeda, Ayala, Regeneron, Inivata, and Novartis. **SAS** is an employee of Guardant Health. **JG, JBR, JCC,** and **PM** are employees of Janssen R&D, and own stock and/or stock options in Johnson & Johnson. **MS** is a former employee of Janssen R&D and own stock and/or stock options in Johnson & Johnson. **NG** reports research/grant support from MS, AstraZeneca, AbbVie, Amgen, Boehringer Ingelheim, Eli Lilly, Hoffmann‐La Roche, Janssen, Merck, MSD, Novartis, Pfizer, Sivan, and Trizell, and consultative services for Bristol Myers Squibb, AstraZeneca, AbbVie, Amgen, Boehringer Ingelheim, Eli Lilly, Hoffmann‐La Roche, Janssen, Merck, MSD, Novartis, Pfizer, Sanofi, and Sivan. A family member is an employee of AstraZeneca.

## Author contributions

SV, AM, LB, S‐HIO, JMB, MS, JG, JBR, JCC, PM, and NG contributed to conceptualization of study. MS, JG, and SAS contributed to data curation. MS and JG contributed to formal analysis and methodology. All authors contributed to writing and reviewing of the manuscript.

### Peer review

The peer review history for this article is available at https://publons.com/publon/10.1002/1878‐0261.13327.

## Supporting information


**Table S1.** EGFR ex20ins variants detected by PCR screening kits.
**Table S2.** EGFR ex20ins variants identified in patients from the GENIE dataset (*N* = 180).
**Table S3.** EGFR ex20ins variants identified in patients from the FoundationInsights dataset (*N* = 627).
**Table S4.** EGFR ex20ins variants identified in patients from the GuardantINFORM dataset (*N* = 627).

## Data Availability

The data sharing policy of Janssen Pharmaceutical Companies of Johnson & Johnson is available at https://www.janssen.com/clinical‐trials/transparency. Data from the AACR Project GENIE are publicly available. Data from Foundation Medicine and Guardant were used under license for the current study and are not publicly available. Other researchers should contact Foundation Medicine and Guardant Health.
