# Supplementary material for: Frequency, underdiagnosis, and heterogeneity of epidermal growth factor receptor exon 20 insertion mutations using real‐world genomic datasets
Source: Mol Oncol. 2022 Nov 28;17(2):230–7. doi: 10.1002/1878-0261.13327 (PMC9892822; doi:10.1002/1878-0261.13327)
Supplement: Supplementary file 1 — Table S1. EGFR ex20ins variants detected by PCR screening kits. Table S2. EGFR ex20ins variants identified in patients from the GENIE dataset (N = 180). Table S3. EGFR ex20ins variants identified in patients from the FoundationInsights dataset (N = 627). Table S4. EGFR ex20ins variants identified in patients from the GuardantINFORM dataset (N = 627). [file MOL2-17-230-s001.docx]

**Supporting information**

**Supplementary Table S1. EGFR ex20ins variants detected by PCR screening kits**.

**Supplementary Table S2. EGFR ex20ins variants identified in patients from the GENIE dataset (N=180)**. Ex20ins variants that were only found in the GENIE database are colored gray.

**Supplementary Table S3. EGFR ex20ins variants identified in patients from the FoundationInsights dataset (N=627)**. Ex20ins variants that were only found in the FoundationInsights database are colored gray.

**Supplementary Table S4. EGFR ex20ins variants identified in patients from the GuardantINFORM dataset (N=627)**. Ex20ins variants that were only found in the GuardantINFORM database are colored gray.

**Supplementary Table S1. EGFR ex20ins variants detected by PCR screening kits.**

| **Mutation** | **Base Change** | **Protein Nomenclature** | **Therascreen** | **Cobas** |
| --- | --- | --- | --- | --- |
| **Ex20ins** | 2307_2308insGCCAGCGTG | A767_V769dup | ✓ | ✓ |
|  | 2319_2320insCAC | H773dup | ✓ | ✓ |
|  | 2310_2311insGGT | D770_N771insG | ✓ | ✓ |
|  | 2311_2312insGCGTGGACA | S768_D770dup | X | ✓ |
|  | 2309_2310AC>CCAGCGTGGAT | A767_V769dup | X | ✓ |

**Supplementary Table S2. EGFR ex20ins variants identified in patients from the GENIE dataset (N=180).** Ex20ins variants that were only found in the GENIE database are colored gray.

| **Ex20ins Variant** | **N** | **Ex20ins Variant** | **N** |
| --- | --- | --- | --- |
| S768_D770dup | 38 | D770_N771insGL | 1 |
| A767_V769dup | 34 | D770_N771insKD | 1 |
| N771_H773dup | 16 | D770_N771insSMD | 1 |
| H773dup | 12 | D770_N771insSTN | 1 |
| D770delinsGY | 8 | D770delinsGTH | 1 |
| P772_H773dup | 8 | D770delinsHN | 1 |
| A763_Y764insFQEA | 5 | H773_V774dup | 1 |
| D770_N771insG | 5 | H773_V774insGNPH | 1 |
| H773_V774insAH | 5 | H773_V774insHH | 1 |
| N771dup | 5 | H773_V774insY | 1 |
| V769_D770insGSV | 4 | H773delinsPNPY | 1 |
| D770_N771insY | 3 | N771_P772insHH | 1 |
| N771_P772insH | 3 | N771_P772insHN | 1 |
| D770_N771insGD | 2 | N771delinsGD | 1 |
| H773_V774insTH | 2 | N771delinsSTH | 1 |
| N771_P772insG | 2 | N771delinsVH | 1 |
| N771_P772insV | 2 | P772_C775dup | 1 |
| P772_H773insGNP | 2 | P772_H773insGDP | 1 |
| P772_H773insYNP | 2 | P772_H773insPNP | 1 |
| A767_S768insIA | 1 | P772_H773insQ | 1 |
| D770_N771insGF | 1 |  |  |

**Supplementary Table S3. EGFR ex20ins variants identified in patients from the FoundationInsights dataset (N=627)**. Ex20ins variants that were only found in the FoundationInsights database are colored gray.

| **Ex20ins Variant** | **N** | **Ex20ins Variant** | **N** |
| --- | --- | --- | --- |
| V769_D770insASV | 137 | D770_N771>GVVDN | 3 |
| D770_N771insSVD | 116 | D770_N771insGF | 3 |
| H773_V774insNPH | 52 | D770_N771insGT | 3 |
| A763_Y764insFQEA | 32 | D770_N771insH | 3 |
| D770_N771insG | 26 | H773_V774insGNPH | 3 |
| H773_V774insH | 26 | N771_P772>GYP | 3 |
| H773_V774insPH | 24 | N771_P772insT | 3 |
| V774_C775insHV | 15 | P772_H773insGNP | 3 |
| H773_V774insAH | 12 | P772_H773insPNP | 3 |
| N771_P772insH | 9 | D770_N771>GSVDN | 2 |
| N771_P772insN | 9 | D770_N771insGD | 2 |
| N771>GY | 8 | D770_N771insGL | 2 |
| H773_V774insY | 7 | D770_N771insY | 2 |
| C775_R776insPHVC | 7 | H773_V774>LM | 2 |
| N771_P772insG | 6 | H773_V774insHPH | 2 |
| N771>GF | 6 | H773_V774insPHPH | 2 |
| S768_V769insVDS | 5 | H773_V774insSH | 2 |
| A767_S768insSVA | 4 | N771_P772>SVDNP | 2 |
| H773_V774insTH | 4 | N771_P772insNN | 2 |
| P772_H773insDNP | 4 | N771_P772insV | 2 |
| S768_V769>IL | 4 | N771_P772insVDN | 2 |
| V769_D770insGSV | 3 | S768_V769>TLASV | 2 |
| A763_Y764insLQEA | 1 | N771_P772>KGP | 1 |
| A767_S768insTLA | 1 | N771_P772>RDP | 1 |
| D770_N771>ASVDS | 1 | N771_P772>SEDNS | 1 |
| D770_N771>EDN | 1 | N771_P772>SHP | 1 |
| D770_N771>EGN | 1 | N771_P772>SPHP | 1 |
| D770_N771>GVMDN | 1 | N771_P772>SVDSP | 1 |
| D770_N771>QVH | 1 | N771_P772insC | 1 |
| D770_N771>RDG | 1 | N771_P772insGTDN | 1 |
| D770_N771insAVD | 1 | N771_P772insHN | 1 |
| D770_N771insGTD | 1 | N771_P772insY | 1 |
| D770_N771insGV | 1 | N771>CH | 1 |
| D770_N771insNPH | 1 | N771>PH | 1 |
| D770_N771insP | 1 | P772_H773>RHPH | 1 |
| D770_N771insSMD | 1 | P772_H773insGCP | 1 |
| D770_N771insSTH | 1 | P772_H773insGDP | 1 |
| D770_N771insT | 1 | P772_H773insNP | 1 |
| H773_V774>NPNPYV | 1 | P772_H773insNPHP | 1 |
| H773_V774>PNPYV | 1 | P772_H773insNV | 1 |
| H773_V774insGH | 1 | P772_H773insQSPNP | 1 |
| H773_V774insNPHPH | 1 | P772>HR | 1 |
| H773_V774insVH | 1 | S768_V769>PL | 1 |
| H773>NPNPY | 1 | S768_V769insMDS | 1 |
| H773>QW | 1 | V769_D770insCV | 1 |
| M793_P794>ITQLMP | 1 | V769_D770insGTV | 1 |
| N771_H773>HHPH | 1 | V769_D770insSASV | 1 |
| N771_P772>HHP | 1 | V769_D770insSLRD | 1 |
| V769_D770insSSV | 1 | V774_C775>GTNPHVC | 1 |
| V769_H773>LDNPNPH | 1 | V774_C775insHNPHV | 1 |
| V769_N771>LDNRGH | 1 | Y764_V765insHH | 1 |

**Supplementary Table S4. EGFR ex20ins variants identified in patients from the GuardantINFORM dataset (N=627)**. Ex20ins variants that were only found in the GuardantINFORM database are colored gray.

| **Ex20ins Variant** | **N** | **Ex20ins Variant** | **N** |
| --- | --- | --- | --- |
| A767_V769dup,V769_D770insASV | 143 | P772delinsHR | 3 |
| S768_D770dup | 117 | V769_D770insGVV | 3 |
| N771_H773dup,H773_V774insNPH | 37 | H773delinsYNPY | 3 |
| A763_Y764insFQEA | 36 | N771delinsHH | 3 |
| H773dup | 33 | H773_V774insY | 2 |
| P772_H773dup | 27 | P772_C775dup | 2 |
| D770delinsGY | 23 | N771_P772insG | 2 |
| D770_N771insG | 20 | N771delinsGF | 2 |
| V774_C775insHV,H773_V774dup | 16 | D770_N771insGT | 2 |
| N771dup | 11 | V769_N771dup | 2 |
| N771_P772insH | 10 | A767_S768insTLA | 2 |
| D770_P772dup,P772_H773insDNP | 8 | H773_V774insGH | 2 |
| N771delinsGY | 7 | H773delinsQW | 2 |
| H773_V774insAH | 6 | V769_D770insCV | 2 |
| P772_H773insPNP | 6 | H773delinsYPNPY | 2 |
| D770_N771insY | 6 | N771_P772insRH | 2 |
| V769_D770insGSV | 4 | N771delinsSH | 2 |
| D770_N771insGF | 4 | S768_V769insVAS | 2 |
| H773_V774insHPH | 4 | V769dup | 2 |
| P772_H773insGNP | 3 | H773delinsSNPY | 2 |
| D770_N771insGL | 1 | D770_V774dup | 1 |
| H773_V774insSH | 1 | D770_N771insTLD | 1 |
| D770_N771delinsRDG | 1 | H773delinsTY | 1 |
| H773delinsNPNPY | 1 | D770_N771dup | 1 |
| N771delinsPH | 1 | V769_D770insGTL | 1 |
| P772_H773insGCP | 1 | V769_D770insGGG | 1 |
| N771_P772dup | 1 | H773_V774insGTNPH | 1 |
| S768_V769insMDS | 1 | V769_D770insNN | 1 |
| V769_D770insSSV | 1 | N771_P772insTHN | 1 |
| V774_C775insHNPHV | 1 | S768_P772dup | 1 |
| Y764_V765insHH | 1 | P772_H773insGHP | 1 |
| P772_H773insYNP | 1 | D770_N771insS | 1 |
| A767_S768insIA | 1 | P772_H773insR | 1 |
| H773delinsPNPY | 1 | H773_V774insAHNPH | 1 |
| H773delinsYQPNPY | 1 | P772_H773insF | 1 |
| D770delinsNNPH | 1 | V774_C775insNPHV | 1 |
| A767_S768insLA | 1 | N771delinsRG | 1 |
| N771delinsKG | 1 | D770delinsGGT | 1 |
| D770delinsEG | 1 | D770_N771delinsAGH | 1 |
| N771_P772insL | 1 | H773delinsNPHPN | 1 |
| P772_H773insQANP | 1 | V769_D770insE | 1 |
| N771_P772insTHTHS | 1 | A767_S768insVA | 1 |
| N771_P772insGG | 1 | H773delinsYY | 1 |
| S768_V769insISS | 1 | H773delinsPYNPY | 1 |
| H773delinsLY | 1 | P772_H773insHPHP | 1 |
